# Ethical and Legal Challenges of Partially and Fully Autonomous AI in Healthcare: Reinterpreting Liability and Preserving Trust

**DOI:** 10.1111/bioe.70137

**Published:** 2026-06-03

**Authors:** Man Teng Iong

**Affiliations:** ^1^ Faculty of Law, University of Macau Macao China

**Keywords:** artificial intelligence, autonomous medical systems, healthcare ethics, liability and accountability, relational trust

## Abstract

Artificial intelligence (AI) is increasingly embedded in healthcare in a variety of ways, ranging from semi‐autonomous decision support systems to the various visions of completely autonomous clinical systems. This article explores the ethical and legal issues that accompany this shift and argues that traditional ways of understanding medical responsibility and the physician‐patient relationships have been strained in light of the increasing autonomy of AI. Ethically, AI raises very acute concerns around transparency and informed consent in the case of “black‐box” systems, the persistence and amplification of bias, privacy risks arising from data‐intensive uses in system development, as well as the harmful effects of automation bias and professional de‐skilling. The article also brings to foreground an underexplored concern—the weakening of relational trust in contexts where clinical judgment and interactions are mediated by an AI, particularly where human oversight of the situation is reduced. Legally, this article outlines challenges of liability across the autonomy spectrum and criticizes proposals that would see AI as an independent liable agent. It promotes a human‐accountability model that includes clinical responsibility for assistive AI, coupled with enterprise measures of liability and post‐market oversight for ever‐more autonomy, backed by mutually appropriate insurance and governance measures. The article ends with some regulatory and institutional recommendations in order to facilitate safe, ethical, and equitable integration of AI in healthcare.

## 1 Introduction

Artificial Intelligence (AI) systems—varying in level of autonomy from decision support tools to hypothetical fully autonomous AI physicians—are transforming healthcare [1, 2]. These technologies promise better diagnostic accuracy, personalization of treatment, and efficiency in the provision of care [3, 4]. However, their adoption involves major ethical and legal questions. As AI is allowed to play a larger role in decision‐making, traditional models of medical responsibility and physician‐patient relationships are questioned. Ethical issues such as transparency, fairness, and consent are acute when AI algorithms act as opaque “black boxes” that play a role in healthcare. Similarly, the legal frameworks have difficulty in allocating responsibility when an AI system is part of a medical error: who is responsible, the physician, the hospital, the software developer or the AI itself?

This article examines the ethical and legal challenges posed by partially and fully autonomous AI in healthcare and gives sustained attention to both dimensions. In particular, it adds two novel points of view: (1) a novel legal argument reinterpreting liability in medical AI to ensure the accountability of human stakeholders, and (2) an under‐discussed ethical concern for the loss of relational trust in AI‐mediated clinical interactions. Throughout, it relies on existing scholarship and policy guidance to suggest the ways in which healthcare might integrate AI systems in a safe, ethical, and responsible way.

## 2 Ethical Challenges in AI‐Mediated Healthcare

AI technologies in healthcare raise a range of ethical problems, some of which are well known and others of which have emerged more recently. Partially autonomous systems (like diagnostic decision support systems of physicians) and fully autonomous systems (like some AI‐driven ability of diagnostic task and surgical assistance of operating with limited human intervention) have overlapping ethical challenges—but to a different degree. Key issues include transparency and informed consent, fairness and bias, patient autonomy, privacy, and the doctor–patient relationship, including the element of trust. Crucially, there appears to be a risk that the introduction of AI (particularly where there is increased autonomy) will lead to a loss of the relational trust that is fundamental to therapeutic alliances.

Transparency and informed consent in medicine require the understanding of the nature and purpose of interventions by patients [5, 6]. AI makes this principle difficult when decision‐making processes are non‐intuitive or opaque. Many AI models (especially deep learning systems) are “black boxes”, producing recommendations that are even impossible to explain in detail for experts [2, 7]. This opacity makes it difficult to explain to the patient why an AI suggested a given diagnosis or treatment. If patients (and even physicians) cannot understand how an AI functions, can patients provide informed consent to AI‐informed care? Lack of transparency can also impact on trust: patients may be reluctant to trust recommendations if they know that such recommendations stem from inscrutable algorithms. It is commonly expressed by experts that making clinical AI explainable is ethically important for maintaining patient understanding and trust [8, 9].

In terms of fairness and biases, AI systems that are trained on healthcare data may inadvertently learn and perpetuate social biases that exist in healthcare data. Bias can occur along lines such race, gender and socioeconomic status or others, and result in unfair or unequal outcomes of care. For example, an algorithm that has been trained mostly on one demographic may not perform well on others which may lead to misdiagnosis or inadequate treatment recommendations for under‐represented groups [10, 11]. This raises issues of justice and equity in healthcare and is a violation of the ethical principle of fairness. Partially autonomous decision aids could give a vigilant physician a chance to pick up and correct a biased recommendation, while a fully autonomous AI operating unstirred by human oversight could be directly perpetuating discrimination. Moreover, biases that are embedded in complex models are often a challenge to identify and fix because of the black‐box nature of advanced AI [12, 13]. Ethically, developers and healthcare institutions have a duty to monitor and reduce bias in the AI tools to avoid further propagating disparities in access to healthcare [14, 15]. Ongoing auditing for the fairness of AI algorithms, as well as using a diverse set of training data, are proposed best practices in upholding fairness [15, 16].

Patient autonomy and privacy can also be affected. AI's hunger for data is leading to privacy concerns that correlate to the issues of autonomy and trust. Large amounts of patient information are used to train and to run AI systems, from electronic health records to imaging information. This raises questions about consent and confidentiality of data. Patients may not fully know that their information is being used to fuel algorithms, especially if AI tools are fully embedded in the patient's care. The Oviedo Convention (1997) and other frameworks assert that the consent of patients should be informed and free for any instance of medical intervention, which by extension includes using patient data and AI involvement [17, 18]. If AI involves patient data outside of the immediate context of care (e.g., for the development of algorithms), this could conflict with privacy rights and data protection legislation. Regulations such as the EU's General Data Protection Regulation (GDPR) define health data as especially sensitive and require very specific conditions for their use [19]. Ensuring that AI is backed up with respect for confidentiality and by appropriate consent (or anonymizing data) is an ethical and legal requirement. In clinical practice, healthcare providers must be transparent about how AI systems utilize patient data and how they are safeguarding it, in order to not betray the trust that patients are placing in the system. A violation of patient data can have a direct negative impact on trust: patients with concerns about how their data will be used can withhold data or refuse care.

Automation bias and professional integrity are also of concern. The introduction of AI decision aids can lead to automation bias—the tendency to over‐rely on the suggestions of an automated system, even when they are apparently incorrect [19, 20]. Physicians could unknowingly defer to the judgment of an AI at the potential cost of their clinical skills, particularly if the AI is said to be very accurate. Over time, routine reliance on AI may lead to a process of de‐skilling of physicians' practice, as they may lose the practice of some skills in diagnosis or decision‐making [21, 22]. This presents an ethical issue of preserving professional competence and critical thinking. In partially autonomous settings, physicians are also expected to be the final arbiter of care, but automation bias could potentially undermine that safeguard. If an error occurs as a result of a physician trusting a flawed AI recommendation, responsibility is thus blurred and the ethical requirement of non‐maleficence is undermined. Healthcare organizations should train physicians to use AI as a tool rather than an infallible oracle by encouraging vigilance and having back‐up plans should AI systems fail [23]. Professional guidelines may have to call out that, although AI can be used to augment clinical practice, it cannot replace human judgment and empathy.

Relational trust in AI‐mediated interactions can also be an ethical concern. The doctor‐patient relationship is built upon trust. Patients expect physicians to act in their best interest competently and with care; physicians expect patients to be honest and to follow treatments. The introduction of AI into this relationship, whether that be as a consultant to the physician or an autonomous agent, complicates these trust‐related dynamics. One issue that has not been examined enough is that of the erosion of relational trust when clinical interactions are mediated or influenced by AI systems. In a partially autonomous scenario, patients, if shown that their physicians relied on an AI to diagnose them, may have questions such as “does my physicians trust me (and their own expertise) so little that they need the guidance of a machine?” or would be afraid that the physicians will blindly follow an algorithm instead of listening to the patients' unique context. Studies have revealed mixed consequences on patient–physician trust when AI is involved. For example, a recent study reported that the use of an AI decision support system did not lead to substantial change in overall trust scores on average, but decreased some patient perceptions of their physicians' competence and integrity—notably among women in high‐risk health scenarios [24]. This means that some patients may be less confident in the abilities of their physicians if AI is involved and may in some way see them as less capable or benevolent if they “outsource” the judgment to a machine.

Fully autonomous AI systems are even more challenging for relationships of trust. In a situation in which an AI entity interacts directly with a patient (e.g., an “AI doctor” chatbot triaging symptoms or even an autonomous robot surgeon performing procedures), the trust between a patient and a human caregiver under the traditional model is absent. Patients may not be comfortable entrusting life‐and‐death decisions to a faceless algorithm, especially without the ability to provide empathy, compassion and a personal connection as a human provider can. Healthcare, as is often pointed out by ethicists, is basically a human‐centred activity based on relationships and dignity rather than a technical service [25]. Over‐reliance on automation and efficiency can depersonalize care and risk to undermine empathy—“the very foundation of care”—, hence corroding trust in the healthcare system [19]. If patients begin to perceive that they are merely interacting with machines instead of receiving human care, their motivation to seek care and to give information may diminish. This erosion of a relational trust can have public health consequences because patients become disengaged from a health system they think is cold or untrustworthy [26]. To mitigate this effect, ethical deployment of AI should ensure a human touch. Even when AI tools are employed, healthcare providers should educate their patients to understand that these tools are intended for enhanced functionality (and not for replacements) of the physician judgment and that the end decisions are made with human oversight. Transparency, with regards to what role AI plays and ways to enable patients to raise their questions or opt‐out, can help maintain trust. Some scholars have suggested that trustworthy AI in healthcare is only possible when systems are designed and operationalized with fundamental principles such as human oversight, fairness, transparency, and accountability, which further maintains the trust of the public [27].

To summarize, there are major ethical challenges associated with partially and fully autonomous AI systems. They need careful balancing of innovation with respect for the patient's rights and values. Ensuring transparency, fighting against bias, safeguarding privacy, and above all upholding humanistic care and trust are paramount. If these ethical issues are not addressed, it seems the promise of AI could be compromised because of loss of patient confidence and moral legitimacy.

## 3 Legal Challenges and Liability in AI‐Driven Healthcare

The legal environment surrounding medical AI is changing, and there is a lot of uncertainty. Traditional healthcare law assumes human actors—licensed professionals and healthcare institutions—are making decisions and can be held responsible for errors. AI disrupts this assumption: if an algorithm is involved in the clinical decision making or making decisions without any human intervention, this complicates the question of liability and accountability. Both partially and fully autonomous systems raise issues of liability, though the stakes are higher the more autonomous the system is. Additionally, regulators and lawmakers must adapt existing safety, effectiveness and privacy frameworks—such as HIPAA, FDA oversight of software as a medical device and the GDPR—to the distinctive characteristics of AI, such as its learning capacity and opacity. This section examines liability concerns for varying degrees of AI autonomy and offers a novel legal argument for guaranteeing human accountability along with other considerations for regulation.

### 3.1 Liability in Partially Autonomous Systems

In current practice, AI often acts as an assistive tool—for example, radiology images are read by an AI, which gives a suggestion for a diagnosis, which is then confirmed or rejected by a radiologist. In such cases, most legal experts consider that the human professional is still responsible for the final decision [28]. AI is perceived as a tool, and physicians' duty of care towards their patients means using (or not using) the tool's advice appropriately [29]. If physicians negligently use a flawed AI recommendation, they (and their employing hospitals) could be the target of a malpractice lawsuit, just as it would have been if the physicians had misread a diagnostic test. Thus, partially autonomous AI does not alleviate liability from physicians, it may even increase the standard of care over time. There is an emerging argument that as certain types of AI tools have become highly accurate or standard in the field, physicians could be found negligent for not taking advantage of AI assistance in a situation where they could have prevented an error [30]. Indeed, commentators have pointed out that if AI becomes part of the “standard of care”, physicians may face liability for ignoring it, placing them in a position where they must choose between relying on the machine and risking malpractice exposure [29]. This prospect also poses ethical concerns, as it may subject physicians to pressure of deferment to AI against their own judgment, knowing that the legal hindsight will judge the decision by AI's capability (a phenomenon sometimes called “routine deferment”) [31, 32]. To deal with partial‐AI situations, legal systems are starting to make it clear that the licensed practitioner is the party responsible. For example, if an AI‐based diagnostic aid is FDA‐authorised and used correctly, the physician who offers the final diagnosis is still expected to confirm results and liable for errors in interpretation [28]. Nonetheless, grey areas remain—for instance, if an AI's error is attributable to a flaw in training data unknown to the physician, he or she might in fairness blame the manufacturer. This leads to the sharing or re‐interpretation of liability.

### 3.2 Liability in Fully Autonomous Systems

As AI technology develops, we can imagine AI systems performing clinical duties with minimal or no human input, such as autonomous AI triage bots making referrals or autonomous robotic surgeons. In such cases, the direct involvement of a human physician is reduced or eliminated, creating the so‐called “accountability gap” [33, 34, 35]. If a fully autonomous AI makes a harmful mistake, according to existing laws who is to blame? The patient cannot sue a machine which has no legal personhood. The natural defendants are therefore the stakeholders behind the system: the manufacturer, the developer, the deploying institution or those responsible for its maintenance. Traditional legal doctrines offer some guidance but are stressed with the characteristics of AI.

One approach (the so‐called “Product Liability”) is to treat AI systems like products. If they do not function properly or generate a negligent output, the manufacturers could be held liable under product liability law (especially if the AI systems are considered as medical devices). Indeed, scholars have proposed to extend product liability to include AI developers for defects in algorithms [36]. If an autonomous diagnostic AI makes a disastrously wrong recommendation due to a coding error or an inadequate data source, this approach will hold the developer or company responsible for releasing a “defective” algorithm. This is consistent with the existing legal principles of product liability for the safety of products. However, a challenge here is the fact that many AI systems are adaptive—they learn and change after being implemented. A system may act in unforeseeable ways not so much because of a developer's original design, but because of how it developed with new data. This creates some confusion about the nature of a “defect” and potentially allows developers to argue that they are not responsible for the outcomes that the AI learned after sale. Nonetheless, regulatory proposals (such as the now‐withdrawn EU AI Liability Directive, 2022 [37, 38]) have attempted to make it easier for patients to claim damages from AI developers, by lowering the burden of proof of how the AI caused harm [39]. Ensuring that developers have malpractice insurance or strict product liability for autonomous AI has been floated as a way of keeping developers accountable [40].

Another model (the so‐called “Institutional Liability” or “Vicarious Liability”) is to assign liability to healthcare institutions using the AI, irrespective of the AI's autonomy. Hospitals already bear vicarious liability for the negligence of their staff. By way of analogy, if a hospital introduces a fully autonomous AI system, then the hospital could be determined to be liable for its actions as if the AI were an employee or instrument of the hospital. This approach provides an incentive for institutions to thoroughly vet and monitor the performance of AI, as the cost of failure will be borne by them. It also ensures that patients have a financially solvent entity to claim against. On the negative, placing all liability on providers could be a disincentive for hospitals to use innovative AI because they are afraid of the legal liability, or could result in excessive monitoring costs. Nevertheless, some legal theorists believe that a non‐delegable duty of care could be imposed on healthcare providers: regardless of the degree of autonomy, the duty to provide safe care cannot be delegated to a machine [41]. Thus, hospitals (and by extension its oversight personnel) should be responsible for the AI's behavior. Current regulatory guidance also seems to lean in this direction as it insists on a “human in the loop”. For instance, the EU AI Act considers most medical AI systems as high‐risk and mandates human oversight of such systems [42]. While this is more a matter of prevention than after‐the‐fact liability, it shows that humans cannot be entirely eliminated from responsibility when it comes to healthcare AI.

A far more controversial idea—AI as a legal entity (the so‐called “Electronic Personhood”)—is to give autonomous AI systems a form of legal personhood, so that such systems could be held liable for their decisions. This concept of an “electronic person” has been debated in the European setting and by legal philosophers. Proponents of the position claim that once AI becomes sufficiently autonomous and complex, our current frameworks (blaming physicians, hospitals, or manufacturers) may not be sufficient to determine where the blame lies, especially if humans truly cannot predict or control the specific actions of AI [43]. Giving AI the legal status of a corporation would potentially enable it to be sued or sanctioned directly. In practice, this might entail placing an autonomous AI under liability insurance or commissioning a fund for compensating victims, and could be “punished” by fines or restrictions (e.g., forced updates or removal from service) when it causes harm. However, the ethical and legal implications of this approach are enormous. Critics point out that the declaration of AI as a legal person creates a moral ambiguity between the human and machine and compromises on principles of human dignity in medicine [43]. It could also be a way to let the human actors off the hook: should the AI is proved responsible, for example, its creators or users could evade accountability. There is discomfort with the idea of “punishing” a non‐sentient‐entity—any penalty ultimately impacts on human stakeholders (owners of the AI systems) or deprives society of a useful tool without the AI itself experiencing consequences. At present, there is still no jurisdiction that has adopted full electronic personhood for AI, and it is an unlikely solution given the pushback of ethicists and legal scholars.

### 3.3 Reinterpreting Liability to Ensure Human Accountability

Considering the above, this article suggests a way to approach this issue legally by reinterpreting and allocating liability in a manner that ensures accountability to human stakeholders, even as AI autonomy increases. The aim is to avoid accountability vacuum or externalization of blame to an algorithm altogether. The argument is that any application of AI in healthcare should be coupled with a clearly assigned responsibility to one or more identifiable human or organizational agents. For partially autonomous AI, this entails reaffirmation of primary liability for the clinical decision on the part of the treating physician—AI recommendations are to be viewed as inputs the physician is answerable for in terms of outcomes. Physicians might be able to defend themselves by demonstrating that an AI was used properly (or that insufficient use of available AI was reasonable), but they are unable to abdicate their role as the custodian of the patient's care. Professional medical societies could release guidelines about the standard of practice when using AI, including expectations of double‐checking AI output and disclosing the use of AI to patients in the consent process.

For fully autonomous AI systems, a combination of strict enterprise liability and mandatory insurance is recommended in this article. Specifically, the entity implementing the AI (i.e., a hospital or a company providing an AI diagnostic service) should be held strictly liable for harm resulting from clinical actions of the AI, irrespective of fault. This ensures that victims are compensated and the burden of risk is on those who stand to benefit from AI deployment (in terms of efficiency or profit). To balance this, and not stifle innovation, those entities can be required or encouraged by law to carry insurance or to contract with the AI manufacturers for indemnification. Meanwhile, the developers themselves should also be held responsible through product liability for defects, and possibly even some ongoing responsibility to update and monitor the safety of the AI in the area. One new legal mechanism could be to impose a “duty of oversight” on AI developers and vendors even after sale: Should an autonomous AI system learn something new from new data, a developer could be required to monitor its performance and step in if it starts to behave in a dangerous way. This type of post‐market surveillance [44] is similar to the pharmacovigilance of drugs. Importantly, this article rejects those solutions which eliminate human being entirely from the equation of accountability. The law should not recognize AI as an independent liable agent; instead, it should strengthen the notion that humans—regardless of if they are software engineers, corporate executives, or clinical directors—should hold themselves accountable for the tools that they created and deployed. Such a position reflects recent policy frameworks such as the US Blueprint for an AI Bill of Rights (2022) [45] and the US Department of Health and Human Services Trustworthy AI Playbook (2021) [46], which call for human accountability in automated systems and protection of patient rights in AI‐mediated care.

Ongoing US legal and regulatory developments are characterized by the intuitive element that AI responsibility should be held by human and institutional actors, as opposed to being relocated to the system itself. The AB 3030 in California mandates disclosure when patient clinical communications are generated using generative AI, and maintains a pathway to a human professional [47]. In greater detail, SB 24‐205 of Colorado imposes on developers and deployers of high‐risk AI systems reasonable care, documentation, impact assessment, and (where technologically possible) human review of adverse consequential decisions [48]. The HTI‐1 final rule at the federal regulatory level entails transparency of predictive decision support intervention (plain‐language information on the developer, training and validation setting, fairness procedures, and external validation) [49], whereas FDA guidance on AI‐enabled medical devices focuses on the lifecycle oversight, planned modification protocols, and continuing performance monitoring [50]. Collectively, these factors indicate a trend toward the legal acceptance of the idea that AI systems cannot work in a responsibility‐free zone.

However, these measures cannot solve the main issue that this article brings up. They consist mainly of ex ante governance and compliance mechanisms, as opposed to a healthcare‐specific model of ex post liability and redress to patients in the event of autonomous clinical AI causing harm. The California statute is confined primarily to disclosure requirements. The model of Colorado is more comprehensive, though it is still a general high‐risk AI law founded on reasonable care, as opposed to enterprise liability, and not designed to address the unique fiduciary and vulnerability aspects of clinical practice. Federal health IT and device regulations also favour transparency, validation, and oversight, but they do not alone establish who is to be legally responsible should injury be incurred. The model offered here, thus, goes a step further, by aligning liability to the extent of autonomy: maintaining physician responsibility to autonomous AI, subjecting the institution that uses autonomous clinical systems to strict enterprise liability, ensuring that compensation mechanisms are backed by insurance, and accepting a residual responsibility of oversight of developers and vendors after implementation. The solution maintains human responsibility in choosing not to fake AI legal personhood and does more to safeguard patient safety and relational trust.

### 3.4 Regulatory Frameworks and Governance

Beyond liability on lawsuits, the challenge is also to update healthcare regulations to ensure safe and effective AI systems. Regulatory bodies like the US FDA and European Medicines Agency are struggling with how to assess AI, especially “adaptive” AI which changes over time. The FDA has started to consider some types of AI algorithms as medical devices (Software as a Medical Device, SaMD) that must be subject to FDA approval and quality standards [51]. The EU AI Act explicitly classifies medical AI as high‐risk, creating requirements for risk management, data management to avoid bias, transparency to users, and human oversight [42]. Such regulations are aimed at preventing harm from occurring and defining responsibilities: developers need to implement accuracy and bias mitigation; providers need to use AI in appropriate and transparent ways. There is also movement towards setting standards for AI explainability in healthcare—that is, requiring that algorithms give reasons for their outputs in understandable terms, at least to physicians, which is in line with ethical calls for transparency [42, 48]. Furthermore, policy documents, such as the WHO's guidance on how to approach ethics in health regarding AI (2021), encourage countries to develop regulations that enshrine a set of principles which include beneficence, non‐maleficence, autonomy, and justice in the context of AI, including measures for data protection and accountability [14].

An additional aspect of governance is providing ongoing oversight. Some have suggested mandatory ethical review boards or committees of AI oversight in hospitals which should audit AI tools periodically for performance and fairness [52]. Internally, healthcare organizations should have clear policies regarding the use of AI—for example, the healthcare organizations should ensure that physicians inform patients when an AI is used in their care, establish protocols to guide what to do if the AI's recommendation contrasts with clinical intuition, and outline who will take responsibility in different scenarios [15, 45]. These “micro‐level” governance measures, which are undertaken by hospitals and professional bodies, complement national and international regulations. By implementing the ethical and legal guardrails at various levels, the healthcare system can manage the risks of AI more effectively and reap its advantages.

## 4 Conclusion

Partially and fully autonomous AI systems in healthcare offer significant promise together with complex ethical and legal issues. Ethically, they make us protect fundamental values—being there to make sure that technology does not erode fairness, transparency, privacy or compassionate trust‐based relationship at the heart of medicine. Legally they call for innovation in our liability doctrines and regulatory regimes so that more AI's autonomy increases, and accountability will not decrease. This article reported on two important aspects: the importance of the re‐interpretation of liability to ensure human stakeholder accountability for AI‐enabled care, and the often‐neglected importance of relational trust within an AI‐enabled clinical environment. A viable future for AI in healthcare is the result of having human agency and responsibility at the centre. No matter how smart or autonomous our machines might be allowed to become, they have to work as caretakers with humans rather than as replacements that lack accountability and empathy. Robust regulatory frameworks—ranging from international laws to hospital policies—should ensure that AI tools are appropriately evaluated, transparently deployed, and constantly monitored. When harms are inflicted, patients deserve explicit opportunities to receive redress, and this means there must be legal fault for those who design, deploy, or oversee AI systems. At the same time, keeping the trust of patients means remembering to make healthcare human‐centric: using AI to enhance human decision‐making and communication, but not replace it. As we enter a time where technological innovation and bioethical tradition converge at their intersection, the guiding principle should be that AI should serve the interests of healthcare to humanity without compromising human accountability nor the human connection. One way that we will need to achieve this balance is with continued interdisciplinary effort—ethicists, legal scholars, physicians, engineers, and policymakers coming together to continue perfecting the way we govern the integration of AI into the healing arts. The present challenges are certainly great, but with careful governance and vigilance for ethical concerns, the potential of AI to support safer, more efficacious, and more equitable healthcare may be realized in a manner that genuinely earns public trust.

## Conflicts of Interest

The author declares no conflicts of interest.

## Data Availability

The author has nothing to report.

## References

[1] S. Maleki Varnosfaderani and M. Forouzanfar , “The Role of AI in Hospitals and Clinics: Transforming Healthcare in the 21st Century,” Bioengineering 11, no. 4 (April 2024): 337, 10.3390/bioengineering11040337. 38671759 PMC11047988

[2] A. I. Padela , R. Hayek , A. Tabassum , F. Jotterand , and J. Qadir , “Assisting, Replicating, or Autonomously Acting? An Ethical Framework for Integrating AI Tools and Technologies in Healthcare,” Bioethics 40, no. 1 (2026): 52–60, 10.1111/bioe.70019. 40679067

[3] D. C. Angus , R. Khera , T. Lieu , et al., “AI, Health, and Health Care Today and Tomorrow: The JAMA Summit Report on Artificial Intelligence,” Journal of the American Medical Association 334, no. 18 (November 2025): 1650–1664, 10.1001/jama.2025.18490. 41082366

[4] P. Rajpurkar , E. Chen , O. Banerjee , and E. J. Topol , “AI in Health and Medicine,” Nature Medicine 28, no. 1 (2022): 31–38, 10.1038/s41591-021-01614-0. 35058619

[5] Council on Ethical and Judicial Affairs , AMA Code of Medical Ethics: 2.1.1 Informed Consent. Chicago, 2016, accessed 16 December 2025, https://code-medical-ethics.ama-assn.org/sites/amacoedb/files/2024-12/2.1.1_0.pdf.

[6] T. L. Beauchamp and J. F. Childress , Principles of Biomedical Ethics, 8th ed. (Oxford University Press, 2019).

[7] C. Rudin , “Stop Explaining Black Box Machine Learning Models for High Stakes Decisions and Use Interpretable Models Instead,” Nature Machine Intelligence 1, no. 5 (May 2019): 206–215, 10.1038/s42256-019-0048-x. PMC9122117 35603010

[8] S. Reddy , “Explainability and Artificial Intelligence in Medicine,” Lancet Digital Health 4, no. 4 (April 2022): e214–e215, 10.1016/S2589-7500(22)00029-2. 35337639

[9] J. Amann , A. Blasimme , E. Vayena , D. Frey , and V. I. Madai , “Explainability for Artificial Intelligence in Healthcare: A Multidisciplinary Perspective,” BMC Medical Informatics and Decision Making 20, no. 1 (December 2020): 310, 10.1186/s12911-020-01332-6. 33256715 PMC7706019

[10] Z. Obermeyer , B. Powers , C. Vogeli , and S. Mullainathan , “Dissecting Racial Bias in an Algorithm Used to Manage the Health of Populations,” Science (1979) 366, no. 6464 (October 2019): 447–453, 10.1126/science.aax2342. 31649194

[11] A. J. Larrazabal , N. Nieto , V. Peterson , D. H. Milone , and E. Ferrante , “Gender Imbalance in Medical Imaging Datasets Produces Biased Classifiers for Computer‐Aided Diagnosis,” Proceedings of the National Academy of Sciences 117, no. 23 (June 2020): 12592–12594, 10.1073/pnas.1919012117. PMC7293650 32457147

[12] R. Challen , J. Denny , M. Pitt , L. Gompels , T. Edwards , and K. Tsaneva‐Atanasova , “Artificial Intelligence, Bias and Clinical Safety,” BMJ Quality & Safety 28, no. 3 (March 2019): 231–237, 10.1136/bmjqs-2018-008370. PMC6560460 30636200

[13] L. H. Nazer , R. Zatarah , S. Waldrip , et al., “Bias in Artificial Intelligence Algorithms and Recommendations for Mitigation,” PLOS Digital Health 2, no. 6 (June 2023): e0000278, 10.1371/journal.pdig.0000278. 37347721 PMC10287014

[14] World Health Organization , “Ethics and Governance of Artificial Intelligence for Health: WHO Guidance,” World Health Organization, 2021, https://iris.who.int/server/api/core/bitstreams/f780d926-4ae3-42ce-a6d6-e898a5562621/content.

[15] M. H. Chin , N. Afsar‐Manesh , A. S. Bierman , et al., “Guiding Principles to Address the Impact of Algorithm Bias on Racial and Ethnic Disparities in Health and Health Care,” JAMA Network Open 6, no. 12 (2023): e2345050, 10.1001/jamanetworkopen.2023.45050. 38100101 PMC11181958

[16] European Commission High‐Level Expert Group on Artificial Intelligence , “Ethics Guidelines for Trustworthy AI,” Brussels, published 2019, https://ec.europa.eu/digital‐.

[17] Council of Europe , “Convention on Human Rights and Biomedicine (Oviedo Convention),” published 1997, https://rm.coe.int/168007cf98.

[18] UNESCO , “Universal Declaration on Bioethics and Human Rights,” published 2005, accessed December 16, 2025, https://unesdoc.unesco.org/ark:/48223/pf0000142825.

[19] S. Capella , “How Does Generative AI Affect Patients' Rights? A Focus on Privacy, Justice, and Autonomy,” Voices in Bioethics 11 (2025): 137–156, 10.52214/vib.v11i.14212.

[20] R. Khera , M. A. Simon , and J. S. Ross , “Automation Bias and Assistive AI Risk of Harm From AI‐Driven Clinical Decision Support,” Journal of the American Medical Association 330, no. 23 (2023): 2255–2257, 10.1001/jama.2022. 38112824

[21] D. Lyell and E. Coiera , “Automation Bias and Verification Complexity: A Systematic Review,” Journal of the American Medical Informatics Association 24, no. 2 (March 2017): 423–431, 10.1093/jamia/ocw105. 27516495 PMC7651899

[22] K. Budzyń , M. Romańczyk , D. Kitala , et al., “Endoscopist Deskilling Risk After Exposure to Artificial Intelligence in Colonoscopy: A Multicentre, Observational Study,” Lancet Gastroenterology & Hepatology 10, no. 10 (October 2025): 896–903, 10.1016/S2468-1253(25)00133-5. 40816301

[23] American Medical Association , “Augmented Intelligence in Medicine,” accessed December 16, 2025, https://www.ama-assn.org/practice-management/digital-health/augmented-intelligence-medicine.

[24] A. G. M. Zondag , R. Rozestraten , S. G. Grimmelikhuijsen , et al., “The Effect of Artificial Intelligence on Patient‐Physician Trust: Cross‐Sectional Vignette Study,” Journal of Medical Internet Research 26 (2024): e50853, 10.2196/50853. 38805702 PMC11167322

[25] M. C. Beach and T. Inui , “Relationship‐Centered Care: A Constructive Reframing,” supplement, Journal of General Internal Medicine 21, no. S1 (January 2006): S3–S8, 10.1111/j.1525-1497.2006.00302.x. PMC1484841 16405707

[26] E. Aranda , L. Ventura Molina , E. Vaquera , E. Matos Pichardo , and O. Iyamu , “Hesitation to Seek Healthcare Among Immigrants in a Restrictive State Context,” Social Sciences 14, no. 7 (July 2025): 433, 10.3390/socsci14070433.

[27] P. Nong and M. Ji , “Expectations of Healthcare AI and the Role of Trust: Understanding Patient Views on How AI Will Impact Cost, Access, and Patient‐Provider Relationships,” Journal of the American Medical Informatics Association 32, no. 5 (May 2025): 795–799, 10.1093/jamia/ocaf031. 40036944 PMC12012342

[28] W. N. Price , S. Gerke , and I. G. Cohen , “Potential Liability for Physicians Using Artificial Intelligence,” Journal of the American Medical Association 322, no. 18 (November 2019): 1765–1766, 10.1001/jama.2019.15064. 31584609

[29] A. Littrell , “The New Malpractice Frontier: Who's Liable When AI Gets It Wrong?,” *Medical Economics* 102 (2025): 8, https://www.medicaleconomics.com/view/the-new-malpractice-frontier-who-s-liable-when-ai-gets-it-wrong-.

[30] S. Gerke , T. Minssen , and G. Cohen , “Ethical and Legal Challenges of Artificial Intelligence‐Driven Healthcare.” Artificial Intelligence in Healthcare (Elsevier, 2020), 295–336, 10.1016/B978-0-12-818438-7.00012-5.

[31] J. J. Hatherley , “Limits of Trust in Medical AI,” Journal of Medical Ethics 46, no. 7 (July 2020): 478–481, 10.1136/medethics-2019-105935. 32220870

[32] M. C. Elish , “Moral Crumple Zones: Cautionary Tales in Human‐Robot Interaction,” Engaging Science, Technology, and Society 5 (March 2019): 40–60, 10.17351/ests2019.260.

[33] A. Matthias , “The Responsibility Gap: Ascribing Responsibility for the Actions of Learning Automata,” Ethics and Information Technology 6, no. 3 (2004): 175–183, 10.1007/s10676-004-3422-1.

[34] E. M. Hille , P. Hummel , and M. Braun , “Meaningful Human Control over AI for Health? A Review,” Journal of Medical Ethics 52, no. e1 (2023): e50–e58, 10.1136/jme-2023-109095. PMC13151516 37730418

[35] K. Prifti , E. Stamhuis , and K. Heine , “Digging Into the Accountability Gap: Operator's Civil Liability in Healthcare AI‐Systems,” in Law and Artificial Intelligence: Regulating AI and Applying AI in Legal Practice, eds. B. Custers and E. Fosch‐Villaronga (T.M.C. Asser Press, 2022), 279–295, 10.1007/978-94-6265-523-2_15.

[36] A. Bertonili , “Artificial Intelligence and Civil Liability,” published 2025, https://www.europarl.europa.eu/RegData/etudes/STUD/2025/776426/IUST_STU(2025)776426_EN.pdf.

[37] European Commission , Proposal for a Directive of the European Parliament and of the Council on Adapting Non‐Contractual Civil Liability Rules to Artificial Intelligence (AI Liability Directive), Brussels, 2022, https://eur-lex.europa.eu/legal-content/EN/TXT/?uri=CELEX:52022PC0496.

[38] European Commission , “Withdrawal of Commission Proposals,” published 2025, https://eur-lex.europa.eu/eli/C/2025/5423/oj.

[39] P. Pirvan , “AI as Product vs. AI as Service: Unpacking the Liability Divide in EU Safety Legislation,” IAPP, accessed December 16, 2025, [Online], https://iapp.org/news/a/ai-as-product-vs-ai-as-service-unpacking-the-liability-divide-in-eu-safety-legislation.

[40] L. McDonald , “AI Systems and Liability: An Assessment of the Applicability of Strict Liability & A Case for Limited Legal Personhood for AI,” St Andrews Law Journal 3, no. 1 (August 2023): 5–21, 10.15664/stalj.v3i1.2645.

[41] G. K. Y. Chan , “AI in Healthcare: Regulatory Guidelines and Judge‐Made Negligence Principles for AI Implementers,” Medical Law International 26, no. 2 (2025): 144–168, 10.1177/09685332251362405.

[42] European Union, Regulation (EU) , 2024/1689 of the European Parliament and of the Council of 13 June 2024 laying down harmonised rules on artificial intelligence (Artificial Intelligence Act), 2024, https://eur-lex.europa.eu/eli/reg/2024/1689/oj/eng.

[43] J. J. Bryson , M. E. Diamantis , and T. D. Grant , “Of, for, and by the People: The Legal Lacuna of Synthetic Persons,” Artificial Intelligence and Law (Dordr). 25, no. 3 (September 2017): 273–291, 10.1007/s10506-017-9214-9.

[44] U.S. Food and Drug Administration , “Proposed Regulatory Framework for Modifications to Artificial Intelligence/Machine Learning–Based Software as a Medical Device,” 2019, https://www.fda.gov/downloads/medicaldevices/deviceregulationandguidance/guidancedocuments/ucm514737.pdf.

[45] White House Office of Science and Technology Policy , “Blueprint for an AI Bill of Rights: Making Automated Systems Work for the American People,” Washington, 2022, https://www.whitehouse.gov/ostp/ai-bill-of-rights.

[46] U.S. Department of Health and Human Services , “Trustworthy AI (TAI) Playbook,” 2021.

[47] California Legislative Counsel , “AB‐3030 Health Care Services: Artificial Intelligence U.S.,” 2024.

[48] Colorado General Assembly , “SB24‐205: Consumer Protections for Artificial Intelligence,” 2024.

[49] Department of Health and Human Services , “Health Data, Technology and Interoperability: Certification Program Updates, Algorithm Transparency, and Information Sharing,” published 2024.

[50] U.S. Food & Drug Administration , “Artificial Intelligence‐Enabled Medical Devices,” 2026.

[51] International Medical Device Regulators Forum , “Software as a Medical Device (SaMD): Clinical Evaluation,” published 2017, https://www.imdrf.org/sites/default/files/docs/imdrf/final/technical/imdrf-tech-170921-samd-n41-clinical-evaluation_1.pdf.

[52] J. Zhang and Z. Zhang , “Ethics and Governance of Trustworthy Medical Artificial Intelligence,” BMC Medical Informatics and Decision Making 23, no. 1 (December 2023): 7, 10.1186/s12911-023-02103-9. 36639799 PMC9840286

